# Features of Serum Fatty Acids at Acute Ischemic Stroke Onset in Statin-Treated Patients with Hypercholesterolemia

**DOI:** 10.3390/nu12092833

**Published:** 2020-09-16

**Authors:** Takahisa Mori, Kazuhiro Yoshioka, Yuhei Tanno, Shigen Kasakura

**Affiliations:** Department of Stroke Treatment, Shonan Kamakura General Hospital, Okamoto 1370-1, Kamakura, Kanagawa 247-8533, Japan; y.kazuhiro12@icloud.com (K.Y.); yxip01@icloud.com (Y.T.); kasakura@med.kitasato-u.ac.jp (S.K.)

**Keywords:** acute ischemic stroke, hypercholesterolemia, fish, seafood, statin, serum fatty acids

## Abstract

In addition to diet therapy, statins are used to prevent cardiovascular disease in patients with hypercholesterolemia (HC). However, acute ischemic stroke (AIS) still occurs in statin-treated patients. How strictly statin-treated patients follow diet therapy before they experience AIS and whether they increase seafood consumption remains unknown. We investigated the serum concentrations and proportions (weight percentages: wt %) of fatty acids (FAs) at AIS onset in statin-treated patients (statin group), compared to those in non-treated patients with HC (6.465 mmol/L or higher) as controls (non-treated group). We included patients with AIS admitted between 2016 and 2019 within 24 h of AIS onset who underwent analysis of serum FAs. During the study period, 188 patients met the inclusion criteria: 133 in the statin group and 55 in the non-treated group. Interestingly, serum FA concentrations in the statin group were lower than those in the non-treated group. However, serum FA wt % in the statin group was almost identical to that in the non-treated group. In conclusion, statin-treated AIS patients had low FA concentrations and identical FA wt %, compared to non-treated AIS patients with HC.

## 1. Introduction

Elevated serum total cholesterol (T-CHO) and triglycerides (TGs) are risk factors for cardiovascular diseases (CVD) [1,2]. In addition to diet therapy, statins are used to lower serum T-CHO levels and prevent coronary heart disease and ischemic stroke [3,4,5]. Dietary guidance focuses on adopting a healthy eating pattern, with an emphasis on consuming fruits, vegetables, legumes, fish, whole grains, low-fat or fat-free dairy products, nuts, seeds, and vegetable oils [6]. There is no specific recommended dietary cholesterol target [7]. Nevertheless, ischemic stroke occasionally occurs in statin-treated patients with hypercholesterolemia (HC). It is unknown why ischemic stroke occurs in these patients despite concomitant diet and statin therapy. Further, how these patients alter their dietary lipid intake derived from meat, vegetables, fish, vegetable oils, dairy products, and seafood is unclear. Dietary TGs influence serum TG and fatty acid (FA) levels [8]. Higher consumption of fish and n-3 polyunsaturated fatty acids (n-3 PUFAs) is associated with a reduced risk of thrombotic infarction [9] and coronary artery disease (CAD) [10] in healthy middle-aged subjects. The weighted percentages (wt %) of individual FAs, with respect to total serum FAs, were previously examined in healthy humans. With increasing age, the wt % of n-6 PUFAs and n-3 PUFAs decrease and increase, respectively [11].

CAD results in increased serum concentrations of palmitic acid (PA), stearic acid (StA), oleic acid (OlA), linoleic acid (LiA), and arachidonic acid (AA), as well as a lower serum concentration of eicosapentaenoic acid (EPA), compared to subjects without CAD. Additionally, the wt % values of EPA and docosahexaenoic acid (DHA) are lower in subjects with CAD than in healthy controls [12]. Administration of highly purified EPA was found to prevent major coronary events and reduce the risk of recurrent stroke in a Japanese population [13,14]. As part of dietary guidance recommendations, patients with HC must change their dietary patterns. In particular, decreased saturated fatty acid (SFA) and n-6 PUFA intake and increased n-3 PUFAs, EPA, and DHA intake are the main goals of diet therapy.

Even with diet therapy, low serum levels and wt % of n-3 PUFAs may persist and be associated with the onset of acute ischemic stroke (AIS). However, serum levels and wt % of FAs before the onset of AIS are unknown. Therefore, we selected patients untreated for HC as controls as they were likely to consume large amounts of SFAs and n-6 PUFAs and small amounts of n-3 PUFAs. Serum FAs at the onset of AIS were examined under non-fasting conditions, and serum FAs under non-fasting conditions are associated with the dietary intake of TGs. In this study, we investigated the serum concentrations and proportions of FAs at admission in statin-treated patients, compared to non-treated AIS patients with HC.

## 2. Materials and Methods

### 2.1. Subjects and Inclusion Criteria

We conducted a cross-sectional study. Our inclusion criteria were patients with AIS who were admitted to our institution between August 2016 and July 2019 within 24 h of AIS onset and who underwent evaluation of blood lipids and FAs at admission. We excluded patients whose (1) serum total cholesterol (T-CHO) level was less than 6.465 mmol/L without statin use, or (2) had a pre-hospital modified Rankin scale score ≥3 or a body mass index <18.5, who were defined as having a severe disability or being underweight, respectively, according to the World Health Organization guidelines. This was done to exclude patients with possible malnutrition.

### 2.2. Measurement of Serum Lipids and FAs

Serum T-CHO, TGs, and high-density lipoprotein-cholesterol (HDL-C) were measured enzymatically using reagents manufactured by Denka Seiken (Denka Co. Ltd., Chuo, Tokyo, Japan) on a BioMajesty 6050 High Throughput Clinical Chemistry Analyzer (JEOL Ltd., Akishima, Tokyo, Japan). Low-density lipoprotein-cholesterol (LDL-C) was calculated using the Friedewald formula as follows: LDL-C = T-CHO − HDL-C − TG/5. We examined the SFAs lauric acid (LaA; C12:0), myristic acid (MyA; C14:0), PA (C16:0), and StA (C18:0); the n-9 MUFA OlA (C18:1); the n-6 PUFAs LiA (C18:2), dihomo-gamma-linolenic acid (DGLA; C20:3), and AA (C20:4); and the n-3 PUFAs alpha-linolenic acid (AlA; C18:3), EPA (C20:5), and DHA (C22:6). We measured the serum concentrations and wt % of each FA at admission. FA concentrations in 1 mL of serum were measured at BML, Inc. (Shibuya, Tokyo, Japan). FAs were extracted according to the general technique of Bligh and Dyer [15], using tricosanoic acid (Nu-Chek Prep, Inc., Elysian, MN, USA) as an internal standard. Lipid extracts were hydrolyzed, extracted with chloroform, and dried under nitrogen gas. After adding a 30% potassium methoxide methanol solution (FUJIFILM Wako Pure Chemical Corporation, Osaka, Osaka, Japan) to the residual sample, it was incubated at 100 °C for 5 min and cooled. Samples were extracted with hexane and analyzed on a GC-2010 Plus Capillary Gas Chromatograph (SHIMADZU Corporation, Kyoto, Japan) equipped with a flame ionization detector, using a BPX70 column (30 m × 0.22 mm ID, 0.25 μm film thickness; SHIMADZU GLC Ltd., Tokyo, Japan). Operating conditions were 50 °C for 0.5 min, followed by a temperature increase to 260 °C over 25 min, which was then maintained for 5 min. Injector and detector temperatures were 240 and 280 °C, respectively, and helium was used as the carrier gas at 1.09 mL/min. Component identification was performed by comparing retention times with those of standards (Sigma-Aldrich Japan, Inc., Meguro, Tokyo, Japan; Nu-Chek Prep, Inc., Elysian, MN, USA). We evaluated the concentrations of serum lipids and FAs and the wt % of serum FAs using internal standard ratios.

### 2.3. Evaluation

We compared patient characteristics, serum lipids, serum concentrations, and proportions of FAs between statin-treated patients with HC (statin group) and non-treated patients with HC (non-treated group). Average blood pressure (ABP) was calculated using the following formula: (systolic blood pressure−diastolic blood pressure)/3 + diastolic blood pressure.

### 2.4. Ethical Approval

All procedures were performed in accordance with the ethical standards of the institution (Shonan Kamakura General Hospital) and the 1964 Helsinki Declaration (the ethical code is TGE01486-024). The Tokusyukai Group Ethical Committee approved our retrospective study.

### 2.5. Consent to Participate

Written informed consent for participation and publication was not required. The study was based on an opt-out model of enrolment, which was permitted by the ethical committee.

### 2.6. Statistical Analysis

Non-normally distributed continuous variables are expressed as medians and interquartile ranges (IQRs). We performed the chi-square test to compare two groups for a categorical variable, and the Wilcoxon rank-sum test to compare non-normally distributed continuous variables between two independent groups: the statin and non-treated group. When there were significant differences in age, sex, lipids levels, and individual FA levels between two groups, we performed multiple logistic regression analysis for lipids and individual FA levels to adjust for age, sex, blood pressure, and group (statin or non-treated). We used general normal limits of the reference range as upper limits of blood lipid levels for multiple logistic regression analysis. On the other hand, the normal reference range for serum FA levels has not yet been determined. When serum FA levels in the statin-treated patients were significantly lower than those in the non-treated patients, we used the first quantile of serum FA levels in the non-treated group as the upper limits of FAs and confirmed whether the group was an independent variable affecting low levels of serum FAs. We reported *p*-values of up to two decimal places if >0.01, three if between 0.001 and 0.01, and “<0.001” otherwise. We presented *p*-values using two-sided tests. JMP software (version 15.1; SAS Institute, Cary, NC, USA) was used for all statistical analyses.

## 3. Results

A total of 880 patients with AIS were admitted to our stroke center during the study period. Among them, 525 patients underwent an examination of serum FAs, and we excluded 340 patients who had serum T-CHO levels of <6.465 mmol/L at admission without having taken statins, or a pre-hospital modified Rankin scale score ≥3, or a body mass index <18.5. A total of 188 patients met our inclusion criteria. Of these 188 patients, 133 patients (25.3%: 133/525) had been taking statins at admission and had undergone statin-treatment for at least 6 months (Table S1). Patient characteristics are summarized in Table 1.

In the statin group, there were more men than women; moreover, patients were older and had lower ABP than those in the non-treated group. Patients in both the groups had glucose intolerance, and there were no differences in serum glucose and HbA1c levels between the two groups. Few patients underwent treatment with antihypertensive drugs in the non-treated group (Table 1). Statin-treated patients had lower serum levels of T-CHO, LDL-C, HDL-C, and TGs. Of note, T-CHO and LDL-C levels were lowered by the well-known statins’ pharmacological effects in statin-treated patients. Further, they also showed lower serum levels of SFAs, n-9 MUFA (OIA), n-6 PUFAs, AlA, and DHA. The wt % of FAs in the statin group, however, were almost the same as those in the non-treated group, except for StA% and LiA% (Table 2). In the statin group, the StA% was higher, and the LiA% was lower, than in the non-treated group. There were no strong correlations between age and ABP (Table S2). After adjusting for age, sex, and ABP by multiple logistic regression, the statin group was an independent variable for decreased concentrations of T-CHO, LDL-C, HDL-C, MyA, PA, StA, OlA, LiA, AA, AlA, and DHA. However, the statin group did not show significant changes in FA wt %. Patients in the statin group had significantly lower concentrations of SFAs, MyA, PA, and StA; n-9 MUFA, OlA, n-6 PUFAs, LiA, and AA; and n-3 PUFAs, AlA, and DHA. The wt % of FAs in the statin group, however, were not significantly different from those of the non-treated group (Table 3, Table 4 and Table 5).

## 4. Discussions

Our results demonstrate that in statin-treated patients, the serum concentrations of lipids and most FAs were lower than those in patients with HC in the non-treated group. The statin group was the most significant independent variable for lowered serum concentrations of lipids and FAs. However, the serum proportions of FAs in the statin group were the same as those in the non-treated group.

In addition to T-CHO and LDL-C, the levels of TGs, SFAs, PA and StA, n-9 MUFA, OlA, n-6 PUFAs, LiA and AA, and n-3 PUFAs (AlA and DHA) were lower in the statin group than in the non-treated group. This may be because of not only statin intake, but also a reduced amount of dietary lipids as an effect of diet therapy for HC. Patients in the statin group may have consumed lower amounts of meat, vegetables, fish, dairy products, vegetable oils, fish oil, and seafood in their diet prior to AIS onset. However, the quality of dietary lipids may have remained unchanged. In this case, the proportions of FAs in the statin group were likely similar to those in the non-treated group.

In the non-treated group, glucose and HbA1c levels were only marginally high, as compared to the statin group, possibly because of an untreated condition. Insufficient therapy for diabetes mellitus was likely associated with AIS onset in the statin group.

The risk of cerebral infarction was 70% lower in patients with T-CHO level <5.68 mmol/L, as compared with patients with T-CHO level >6.20 mmol/L [3]. In our study, the median T-CHO levels in the statin and non-treated groups were 4.50 and 7.01 mmol/L, respectively. However, AIS occurred in the statin group. Therefore, in dietary interventions for HC, lowering T-CHO levels as well as changing the quality of dietary lipids may be equally essential to prevent ischemic stroke.

It has been suggested that n-3 PUFAs can reduce the incidence of CAD and stroke, as well as the mortality associated with cardiovascular diseases [16,17,18,19]. Administration of highly purified EPA appeared to reduce the risk of recurrent stroke in Japanese patients with HC who were administered a low-dose statin therapy [14]. In contrast, SFAs may increase the risk of these conditions [20]. According to dietary guidance, statin-treated patients must change their dietary patterns. A decrease in SFAs and n-6 PUFAs and an increase in n-3 PUFAs, particularly EPA and DHA, are the main goals of such dietary interventions. The diets followed by statin-treated patients in our study contained reduced amounts of dietary lipids but did not change the overall fatty acid proportions. The serum wt % of n-3 PUFAs, particularly EPA and DHA, were not increased and may be associated with the onset of AIS. If patients in the statin group reduced dietary lipid levels as well as increased their EPA and DHA proportions, their risk of AIS occurrence may have been lower [14]. Therefore, to prevent AIS in patients with HC, it may be necessary to increase the dietary intake of EPA and DHA to increase their relative proportions in overall dietary lipid consumed.

In a study of healthy Canadians in their 20s, the median concentrations of LiA, DGLA, EPA, and DHA were 2208.2, 68.2, 32.4, and 82.0 μmol/L, respectively [21], which are all lower than the concentrations detected in our study. Particularly, the median EPA concentrations in our statin and non-treated groups were 182.7 and 261.5 μmol/L, respectively. The median concentrations of DHA in these groups were 355.98 and 468.46 μmol/L, respectively. Notably, the serum levels of EPA and DHA were considerably higher than those in the healthy young cohort. In a study of patients with CAD in the United States with an average age of 47 years, the wt % of PA, StA, OlA, LiA, DGLA, AA, EPA, and DHA were 11.3%, 0.2%, 18.4%, 58.6%, 0.4%, 9.4%, 0.9%, and 0.5%, respectively [12]. The proportions of LiA and AA were higher in US patients than in our groups; however, the proportions of OlA, EPA, and DHA were lower than those in both of our groups. The concentrations and proportions of EPA and DHA in our patients were higher than those in both healthy young Canadians and US patients; however, AIS still occurred in our patients.

Dietary intake of TGs, found in meat, fish, vegetables, and their oils, influences serum FA concentrations and proportions. Therefore, it is essential to assess the types and quantities of dietary TG sources consumed during the days leading up to AIS onset. The development of dietary regimens that change dietary patterns, decrease SFAs and n-6 PUFAs, and increase EPA and DHA concentrations and proportions may contribute to the prevention of AIS.

Our study had several limitations. A small number of patients were included, and the study was retrospective and cross-sectional. As all our patients were East Asians, there may be racial differences in the appropriate concentrations and proportions of EPA and DHA for preventing CVD or ischemic stroke. To determine the effects of EPA or DHA concentrations and proportions on AIS prevention in statin-treated patients, a prospective randomized control study using a food frequency questionnaire (FFQ) and serum FA levels is required.

## 5. Conclusions

Statin-treated AIS patients had low FA concentrations and identical FA wt %, compared to non-treated AIS patients with HC.

## Figures and Tables

**Table 1:** Differences in patient characteristics between statin and non-treated groups.

Variables	Statin Group (*n* = 133)	Non-Treated Group (*n* = 55)	*p*
Age (MD, IQR) (Min, Max) years	**75** (70.5–82.5) (Min: 52, Max: 99)	71 (61–80) (Min: 39, Max: 97)	<0.001
Male sex	**85** **(63.9%)**	21 (38.2)	0.0012
Height (MD, IQR) cm	162 (153.8–168)	156 (150–165)	0.06
Body weight (MD, IQR) kg	60 (53–67.9)	58 (50.6–68)	0.27
Body mass index (MD, IQR) kg/m^2^	23.1 (21.1–25.4)	22.9 (21.0–25.4)	0.95
Glucose and lipids			
Glucose (MD, IQR) mmol/L	6.72 (5.91–8.22)	7.72 (6.00–9.77)	0.051
Hemoglobin A1c (HbA1c) (MD, IQR) % (NGSP)	6.0 (5.7–6.6)	6.1 (5.7–7.6)	0.34
Total cholesterol (MD, IQR) mmol/L	4.50 (4.03–5.09)	**7.01** (6.72–7.47)	<0.001
High-density lipoprotein-cholesterol (HDL-C) (MD, IQR) mmol/L	1.42 (1.19–1.72)	**1.52** (1.33–1.87)	0.04
Triglycerides (MD, IQR) mmol/L	1.21 (0.85–1.69)	**1.65** (1.22–3.06)	<0.001
Low-density lipoprotein-cholesterol (LDL-C) (MD, IQR) mmol/L	2.42 (1.97–3.02)	**4.41** (4.04–5.10)	<0.001
ABP (MD, IQR) mmHg	109 (99.5–117)	**125** (108–141)	<0.001
Antihypertensive drugs	**96 (72%)**	15 (27%)	<0.001

ABP: average blood pressure, Min: minimum, Max: maximum, MD: median, Statin group: statin-treated patients with hypercholesterolemia, non-treated group: patients with hypercholesterolemia not treated with statins; IQR: interquartile range; NGSP: National Glycohemoglobin Standardization Program; wt %: weight percentage of total fatty acids; n-3 PUFA: n-3 polyunsaturated fatty acid; n-6 PUFA: n-6 polyunsaturated fatty acid; n-9 MUFA: n-9 monounsaturated fatty acid; Bold values denote statistical significance at the *p* < 0.05 level.

**Table 2:** Differences in fatty acids between statin and non-treated groups.

Fatty Acids	Statin Group (*n* = 133)	Non-Treated Group (*n* = 55)	*p*
Saturated fatty acids			
Lauric acid (LaA) (MD, IQR) μmol/L	4.49 (2.99–7.98)	4.49 (2.99–10.48)	0.66
Myristic acid (MyA) (MD, IQR) μmol/L	64.39 (50.59–93.08)	**91.54** (65.26–122.2)	<0.001
Palmitic acid (PA) (MD, IQR) μmol/L	2337 (1993–2825)	**3046** (2771–3694)	<0.001
Stearic acid (StA) (MD, IQR) μmol/L	612.8 (531.9–714.9)	**781.4** (682.2–886.0)	<0.001
LaA (MD, IQR) wt %	0 (0–0.1)	0 (0–0.1)	0.26
MyA (MD, IQR) wt %	0.6 (0.5–0.7)	0.6 (0.5–0.8)	0.58
PA (MD, IQR) wt %	23.4 (22.5–24.4)	23.6 (22.7–24.9)	0.31
StA (MD, IQR) wt %	**6.8** (6.2–7.3)	6.6 (5.9–7)	0.02
n-9 MUFA			
Oleic acid (OlA) (MD, IQR) μmol/L	1950 (1576–2474)	**2555** (2180–3110)	<0.001
OlA (MD, IQR) wt %	21.5 (19.8–23.7)	21.4 (19.6–23.7)	0.64
n-6 PUFAs			
Linoleic acid (LiA) (MD, IQR) μmol/L	2312 (2011–2659)	**3126** (2824–3667)	<0.001
Dihomo-gamma-linolenic acid (DGLA) (MD, IQR) μmol/L	87.69 (69.60–110.35)	**125.18** (88.02–157.46)	<0.001
Arachidonic acid (AA) (MD, IQR) μmol/L	536.0 (454.1–603.5)	**674.7** (599.9–805.9)	<0.001
LiA (MD, IQR) wt %	25.3 (22.6–26.7)	**26.3** (23.4–28.5)	0.03
DGLA (MD, IQR) wt %	1 (0.9–1.2)	1.1 (0.9–1.2)	0.54
AA (MD, IQR) wt %	6.3 (5.5–7.3)	6.1 (5.3–6.8)	0.21
n-3 PUFAs			
Alpha-linolenic acid (AlA) (MD, IQR) μmol/L	56.0 (42.0–82.0)	**84.37** (65.34–100.88)	<0.001
Eicosapentaenoic acid (EPA) (MD, IQR) μmol/L	182.7 (142.2–305.0)	261.5 (152.9–344.2)	0.20
Docosahexaenoic acid (DHA) (MD, IQR) μmol/L	355.98 (294.88–441.86)	**468.46** (386.1–572.1)	<0.001
AlA (MD, IQR) wt %	0.6 (0.5–0.8)	0.7 (0.6–0.8)	0.18
EPA (MD, IQR) wt %	2.2 (1.7–3.5)	2.1 (1.1–3.2)	0.09
DHA (MD, IQR) wt %	4.5 (3.9–5.4)	4.4 (3.6–5.6)	0.65
EPA/AA ratio (MD, IQR)	0.34 (0.26–0.58)	0.33 (0.2–0.51)	0.15
n-6/n-3 ratio (MD, IQR)	4.12 (3.14–5.14)	4.54 (3.09–6.06)	0.13

MD: median, statin group: statin-treated patients with hypercholesterolemia, non-treated group: patients with hypercholesterolemia not treated with statins; IQR: interquartile range; wt %: weight percentage of total fatty acids; n-3 PUFA: n-3 polyunsaturated fatty acid; n-6 PUFA: n-6 polyunsaturated fatty acid; n-9 MUFA: n-9 monounsaturated fatty acid; Bold values denote statistical significance at the *p* < 0.05 level.

**Table 3:** Multiple logistic regression analysis for low lipids levels adjusted to age, sex. ABP, and group.

Variables	*N*	Odds Ratio (95% CI)	*p*	AUC
Total cholesterol ≤5.66 mmol/L	188			0.936
group (1: statin, 0: no statin)		**4.82 × 10^10^ (0–∞)**	**<** **0.** **001**	
ABP		0.98 (0.95–0.1.01)	0.15	
Sex (1: male, 0: female)		4.21 (1.40–12.67)	0.009	
age		1.05 (0.99–1.11)	0.13	
Low-density lipoprotein-cholesterol ≤3.6 mmol/L	188			0.911
group (1: statin, 0: no statin)		**103.1 (31.1** **–** **451.3)**	**<0.001**	
ABP		1.00 (0.97–1.03)	0.85	
Sex (1: male, 0: female)		5.88 (2.08–19.39)	0.001	
age		0.99 (0.94–1.06)	0.98	
Triglycerides ≤1.69 mmol/L	188			0.694
group (1: statin, 0: no statin)		1.45 (0.67–3.12)	0.35	
ABP		0.97 (0.95–0.99)	0.003	
Sex (1: male, 0: female)		0.95 (0.47–1.91)	0.89	
age		1.03 (0.99–1.68)	0.06	
High-density lipoprotein-cholesterol ≤1.74 mmol/L	188			0.663
group (1: statin, 0: no statin)		**2.43** **(1.10–5.35)**	**0.03**	
ABP		1.01 (0.99–1.03)	0.47	
Sex (1: male, 0: female)		1.91 (0.95–3.84)	0.07	
age		0.98 (0.94–1.01)	0.19	

ABP: average blood pressure; AUC: area under the curve; CI: confidence interval; Bold values denote statistical significance of the group at the *p* < 0.05 level.

**Table 4:** Multiple logistic regression analysis for low levels of SFAs and n-9 MUFA adjusted to age, sex. ABP, and group.

	*N*	Odds Ratio (95% CI)	*p*	AUC
Myristic acid ≤65.3 μmol/L	188			0.652
group (1: statin, 0: no statin)		**2.53 (1.17–5.45)**	**0.02**	
ABP		0.98 (0.97–1.00)	0.07	
Sex (1: male, 0: female)		0.99 (0.53–1.88)	0.99	
age		1.00 (0.97–1.03)	0.96	
Palmitic acid ≤2771 μmol/L	188			0.797
group (1: statin, 0: no statin)		**6.80 (2.92** **–** **15.80)**	**<0.001**	
ABP		0.96 (0.94–0.98)	<0.001	
Sex (1: male, 0: female)		0.50 (0.23–1.07)	0.07	
age		1.01 (0.98–1.05)	0.47	
Stearic acid ≤682.2 μmol/L	188			0.773
group (1: statin, 0: no statin)		**4.29 (1.96–9.43)**	**<0.001**	
ABP		0.97 (0.95–0.99)	0.001	
Sex (1: male, 0: female)		0.80 (0.40–1.62)	0.54	
age		1.02 (0.99–1.06)	0.16	
Oleic acid ≤2180 μmol/L	188			0.777
group (1: statin, 0: no statin)		**4.34 (1.91–9.84)**	**<0.001**	
ABP		0.97 (0.95–0.99)	<0.001	
Sex (1: male, 0: female)		0.51 (0.25–1.04)	0.06	
age		1.03 (0.99–1.06)	0.11	

ABP: average blood pressure; AUC: area under the curve; CI: confidence interval; MUFA: monounsaturated fatty acid; SFA: saturated fatty acid; Bold values denote statistical significance of the group at the *p* < 0.05 level.

**Table 5:** Multiple logistic regression analysis for low levels of n-6 PUFAs and n-3 PUFAs and FA wt % adjusted to age, sex. ABP, and group.

	*N*	Odds Ratio (95% CI)	*p*	AUC
Linoleic acid (LiA) ≤2824 μmol/L	188			0.832
group (1: statin, 0: no statin)		**8.81 (3.88–20.01)**	**<0.001**	
ABP		0.97 (0.95–0.99)	0.010	
Sex (1: male, 0: female)		1.16 (0.52–2.59)	0.71	
Age		1.05 (1.01–1.10)	0.009	
Dihomo-gamma-linolenic acid ≤88.0 μmol/L	188			0.716
group (1: statin, 0: no statin)		2.01 (0.91–4.45)	0.08	
ABP		0.97 (0.95–0.99)	<0.001	
Sex (1: male, 0: female)		0.88 (0.45–1.70)	0.70	
Age		1.03 (0.99–1.06)	0.14	
Arachidonic acid ≤599.9 μmol/L	188			0.790
group (1: statin, 0: no statin)		**6.27 (2.84–13.85)**	**<0.001**	
ABP		0.99 (0.98–1.01)	0.57	
Sex (1: male, 0: female)		1.73 (0.85–3.49)	0.13	
age		1.04 (1.01–1.08)	0.02	
Alpha-linolenic acid ≤65.3 μmol/L	188			0.729
group (1: statin, 0: no statin)		**3.34 (1.53–7.32)**	**0.002**	
ABP		0.97 (0.95–0.99)	0.003	
Sex (1: male, 0: female)		1.12 (0.58–2.16)	0.73	
age		1.01 (0.97–1.04)	0.74	
Docosahexaenoic acid ≤386.1 μmol/L	188			0.699
group (1: statin, 0: no statin)		**3.88 (1.79–8.42)**	**<0.001**	
ABP		0.98 (0.96–0.99)	0.04	
Sex (1: male, 0: female)		1.18 (0.62–2.26)	0.61	
age		0.99 (0.96–1.02)	0.58	
Stearic acid wt % ≤5.9%	188			0.613
group (1: statin, 0: no statin)		**0.39 (0.16–0.97)**	**0.04**	
ABP		1.00 (0.98–1.02)	0.99	
Sex (1: male, 0: female)		1.10 (0.49–2.48)	0.82	
Age		1.02 (0.98–1.06)	0.29	
LiA wt % ≤23.4%	188			0.603
group (1: statin, 0: no statin)		2.04 (0.90–4.61)	0.08	
ABP		1.02 (1.00–1.04)	0.04	
Sex (1: male, 0: female)		0.86 (0.44–1.69)	0.68	
Age		0.99 (0.96–1.08)	0.73	

ABP: average blood pressure; AUC: area under the curve; CI: confidence interval; FA: fatty acids; PUFA: polyunsaturated fatty acid; Bold values denote statistical significance of the group at the *p* < 0.05 level.

## Supplementary Materials

The following are available online at https://www.mdpi.com/2072-6643/12/9/2833/s1, Table S1: Statins, Table S2: Spearman correlation coefficient between age and average blood pressure.
